# Understanding the social determinants of health among Indigenous Canadians: priorities for health promotion policies and actions

**DOI:** 10.3402/gha.v8.27968

**Published:** 2015-07-16

**Authors:** Fariba Kolahdooz, Forouz Nader, Kyoung J. Yi, Sangita Sharma

**Affiliations:** 1 Aboriginal and Global Health Research Group, Division of Endocrinology and Metabolism, Department of Medicine, Faculty of Medicine and Dentistry, University of Alberta, Edmonton, AB, Canada; 2 School of Human Kinetics and Recreation, Memorial University of Newfoundland, St. John’s, NL, Canada

**Keywords:** social determinants of health, health disparity, systematic review, Indigenous peoples, Canada

## Abstract

**Background:**

Indigenous Canadians have a life expectancy 12 years lower than the national average and experience higher rates of preventable chronic diseases compared with non-Indigenous Canadians. Transgenerational trauma from past assimilation policies have affected the health of Indigenous populations.

**Objective:**

The purpose of this paper is to comprehensively examine the social determinants of health (SDH), in order to identify priorities for health promotion policies and actions.

**Design:**

We undertook a series of systematic reviews focusing on four major SDH (i.e. income, education, employment, and housing) among Indigenous peoples in Alberta, following the protocol *Preferred Reporting Items for Systematic Reviews and Meta-Analysis-Equity*.

**Results:**

We found that the four SDH disproportionately affect the health of Indigenous peoples. Our systematic review highlighted 1) limited information regarding relationships and interactions among income, personal and social circumstances, and health outcomes; 2) limited knowledge of factors contributing to current housing status and its impacts on health outcomes; and 3) the limited number of studies involving the barriers to, and opportunities for, education.

**Conclusions:**

These findings may help to inform efforts to promote health equity and improve health outcomes of Indigenous Canadians. However, there is still a great need for in-depth subgroup studies to understand SDH (e.g. age, Indigenous ethnicity, dwelling area, etc.) and intersectoral collaborations (e.g. community and various government departments) to reduce health disparities faced by Indigenous Canadians.

Indigenous peoples in Canada represent 4.3% of the total Canadian population (1). Indigenous Canadians have a life expectancy 12 years lower than the national average and experience higher rates of preventable chronic diseases compared with non-Indigenous Canadians (2, 3). The causes of these disparities are complex, and it is essential to note that the contextual (e.g. colonization, residential schooling, the resulting trauma, etc.), and ecological circumstances of social determinants of health (SDH) have an important impact on the health and well-being of Indigenous populations (4, 5).

Understanding the context of SDH in Canadian Indigenous peoples, requires examining Canadian history after the 1867 Indian Act. The Act (6) facilitated and enforced the assimilation of Indigenous peoples into European culture by various means: turning Indigenous peoples into wards of the state; replacing traditional governance system with federally imposed reserve systems; forcing Indigenous peoples to give up ‘status’ and heritage. Indigenous children were taken away from families and placed into residential schools and non-Indigenous foster families (7–9). Until 1996, a total of 130 residential schools operated throughout Canada and restricted children from practicing traditional values, ancestral culture, and language through a means of physical penalties. Interacting with their families and communities was often forbidden. Many children experienced severe physical, psychological, spiritual, and sexual abuse. When students left residential schools at the age of 16 years, many settled off-reserve because of the implanted negative perceptions of their heritage and difficulties of coping with traditional life on reserves (7–9). Today, approximately 86,000 people who attended these schools are still alive (8). Many Indigenous adults who were deemed ‘unfit’ to have children were subject to forced sexual sterilization (7–9). These forced assimilation policies have adversely affected Indigenous spirituality, traditions, cultures, languages, and connections to the lands, which are vital to Indigenous well-being and health (7, 10) and often violated the dignity and autonomy of many Indigenous peoples (7). Consequently trauma (damage to an individual due to a psychologically and emotionally overwhelming event or series of severe events) (11) and its continuing effects, have contributed to many present health issues such as mental illness, depression, suicide, violence, and sexual, alcohol, and drug-related vulnerabilities (12–16). For decades, these effects of trauma have been sustained as a form of *transgenerational trauma*, ‘a collective complex trauma inflicted on a group of people who share a specific group identity or affiliation—ethnicity, nationality, or religious affiliation [and] the legacy of numerous traumatic events a community experiences over generations’ (17). Transgenerational trauma is usually embedded within a peoples’ cultural memory, passed on as an integral component of a peoples’ culture, and ‘normalized’ in that culture (7, 8, 10, 13, 18, 19). Many Indigenous Canadian populations have experienced the cycle of transgenerational trauma (8, 20, 21) and cultural, familial, and social dissociation which have influenced material, socioeconomic, and socioenvironmental disparities between Indigenous and non-Indigenous Canadians through generations (7, 9, 20, 22, 23).

The *Integrated Life Course and Social Determinants Model of Aboriginal Health* (24) conceptualizes SDH into three categories: *proximal, intermediate*, and *distal. Proximal* SDH ‘have a direct impact on physical, emotional, mental, or spiritual health’ of Indigenous Canadians (24) [e.g. employment and income (24, 25), education (24, 25), physical housing environments (26), individuals’ health behaviors (27), and food security (28)] and may be more easily targeted by policy changes compared to intermediate (e.g. resources, infrastructure, and opportunities) or distal (e.g. healthcare systems, educational systems, natural environment, cultural continuity, colonialism, and racial discrimination) SDH.

Traditionally, health was simply an absence of illness, but it has long been conceptualized through a more holistic definition that recognizes the importance of physical, psychological, social, and spiritual well-being (29, 30). This concept of health is especially significant for Indigenous health, which refers to preserving physical, mental, emotional, and spiritual well-being and connecting to one's family and community (31). However, the progression towards integrating this concept into policy, and the health agenda is complicated (29). Therefore, SDH should be comprehensively examined to identify priorities for health policies and actions that address the health disparities among Indigenous Canadians. Both federal and provincial/territorial governments of Canada share responsibility for social and health policies (32); yet, there is scarce information on SDH at the provincial/territorial level. This study will undertake a series of systematic reviews focusing on four major SDH: income, education, employment, and housing, to examine the current socioeconomic status and material circumstances facing Indigenous Canadians in the province of Alberta. The authors acknowledge other SDH such as food insecurity and social capital. Nonetheless, we will focus on these four SDH because the Community Well-Being Index of Canada, which assesses socioeconomic well-being of the Indigenous Canadian population at the community level, is made up of these four indices (33).

Alberta has the third largest Indigenous population in Canada, and 6% of the province's total population self-identifies as Indigenous (1). Our systematic review may highlight knowledge gaps and areas requiring further investigation; provide empirical knowledge for future policies and actions at the provincial level; and inform efforts to promote health equity and improve health outcomes of Indigenous Canadians.

## Methods

The systematic review followed the protocol, *Preferred Reporting Items for Systematic Reviews and Meta-Analyses with a focus on health Equity (PRISMA-Equity)* (34). We systematically searched English literature published between January 2000 and August 2014 in four electronic databases (i.e. PubMed, EMBASE, CINAHL, and PsycINFO) and in *Pimatisiwin: A Journal of Aboriginal and Indigenous Community Health*. Grey literature (i.e. informally published written documents such as governmental reports) was identified by 1) searching websites including Public Health Agency of Canada (www.phac-aspc.gc.ca), Statistics Canada (www.statcan.gc.ca), Health Canada (www.hc-sc.gc.ca), National Aboriginal Health Organization (www.naho.ca), The First Nations Information Governance Centre (www.fnigc.ca), and Alberta Health (www.health.alberta.ca); 2) consulting online search engines (Google and Google Scholar); 3) examining direct communications with Indigenous-related agencies and institutes or independent individuals; and 4) reviewing manually the reference lists of selected sources. Search descriptors were the MeSH (Medical Sub-Heading) terms including: “Aboriginal”, “American Native”, “American Native continental ancestry group”, “American Indian”, “Inuit”, “First Nations”, and “Métis”, combined with “Canada” and “Alberta” as well as the MeSH synonyms for each SDH; “income”, “poverty”, “socioeconomic factors”, “education”, “employment”, and “housing”.

Two reviewers (FK, FN) screened the titles and abstracts of all identified sources to remove duplicates and irrelevant records. The reviewers then evaluated the full-text of selected sources independently. Inclusion criteria (e.g. documents written in English and topics related to SDH and Indigenous populations) and the quality appraisal criteria for observation and/or intervention studies (e.g. clear presentation of research goal, participants, methods, and results) were used to ensure inter-reviewer reliability (35). Publications which focused on Indigenous populations at either the national level or the provincial level outside the province of Alberta were excluded. We summarized the most up-to-date census information to provide absolute differences in the four SDH between non-Indigenous peoples in Alberta, Indigenous peoples in Alberta, and Indigenous peoples in Canada (Table 1). Lastly, we extracted information such as first author's name, population group, participants, and outcomes from the selected sources (Table 2). We also identified PROGRESS-Plus factors (Place of residence, Race/ethnicity/culture/language, Occupation, Render, Religion, Education, Socioeconomic Status, Social Capital) (34) to determine which of these factors might have affected the SDH outcomes for Indigenous peoples' health. Then, each study was categorized by theme (i.e. one of the four SDH of interest) and the results were summarized under each thematic category.

**Table 1:** Status of the four major components (income, education, employment, and housing) of social determinants of health in Alberta and Canada

	Non-Indigenous peoples in AB	Indigenous peoples in AB	Indigenous peoples in CA
**Income**			
Average income, $ (2011)			
Total	50,956	35,437	29,780
Male	64,260	43,601	33,570
Female	37,439	27,871	26,341
After-tax average income, $ (2011)			
Total	41,962	30,525	26,258
Male	51,587	36,395	28,871
Female	32,179	25,086	23,887
Average employment income, $ (2011)			
Total	69,438	55,668	48,534
Male	80,112	63,933	54,066
Female	53,952	45,236	42,572
Average householdincome, $ (2011)	100,819	83,437	66,513
After-tax average household income, $ (2011)	83,011	70,625	57,754
Prevalence of low income (%) (2011)			
Total	10.7	20.6	25.3
Male	10.0	18.2	23.5
Female	11.4	22.9	26.8
Composition of total income (%) (2011)			
Earnings	91.1	85.9	80.1
Government	7.3	12.9	18.5
Others	1.6	1.2	1.4
**Employment**			
Participation rate (%)			
2006	74.0	68.3	63.0
2011			
Total	73.7	67.7	61.3
Male	79.8	76.2	64.7
Female	67.3	59.8	58.1
Unemployment rate (%)			
2006	4.3	11.1	14.8
2011			
Total	5.5	11.1	15.0
Male	5.6	12.7	16.8
Female	5.3	9.2	13.3
Employment rate (%)			
2006	70.9	60.8	53.7
2011			
Total	69.7	60.2	52.1
Male	75.3	66.5	53.9
Female	63.7	54.3	50.4
**Housing**			
Dwelling requiring major repairs (%)			
2006	6.7	16.9	18.8
2011	6.4	16.2	17.4
Crowded dwelling (%)			
2006	1.3	4.2	4.2
2011	1.7	4.2	4.1
Spending ≥30% incomeon dwelling (%) (2011)	38.6	43.4	40.8
**Education**			
No high school completion (%)			
2006	22.3	44.3	43.6
2011	17.9	39.8	38.0
High school certificate or equivalent (%)			
2006	26.4	21.4	21.8
2011	26.6	24.2	23.9
Some form of post-secondary certificate ordegree (%)			
2006	51.3	34.3	34.6
2011	55.5	36.0	38.1
University degree (%) (2011)	21.7	6.2	7.4

AB: Alberta; CA: Canada; Sources: (36–43).

**Table 2:** General characteristics of the studies included in the systematic review on four major social determinants of health (income, education, employment, and housing) among Indigenous populations in Alberta

First author's name reference	Study participants, outcomes, and PROGRESS-Plus factor	Summary of results
Income		
Lachance (4)	• First Nations communities in Alberta (*n*=45) • Median after-tax income by First Nations community, educational attainment, and family status, and composition of income • Place of residence, race/ethnicity/culture/language, education and Plus (marital status)	There was a significant gap in median after-tax household income between First Nations households and non-First Nations households in Alberta and Canada. Geography plays an important role in the difference in median after-tax household income among First Nations communities. Median after-tax household income ranged $17,056–$34,176 among Treaty 6 communities; $17,920–$27,456 among Treaty 7 communities; and $22,528–$42,485 among Treaty 8 communities ($50,000–$60,000 among southern and central non-First Nations communities and up to $97,483 among northern non-First Nations communities). Individuals with high school diploma have 50% higher median income compared to those without high school diploma. First Nations individuals with university education may have up to $750,000 greater lifetime earnings compared to those who have not completed high school. Married-couple First Nations households have the highest median after-tax household income compared to common-law couples or single parents. The proportion of government transfers is larger in First Nations in Alberta (18.6%) compared to non-First Nations in Alberta (<10%).
Smoyer-Tomic (44)	• General population including Indigenous peoples in residential areas in a city in Alberta (*n*=215) • The association between neighborhood socioeconomic status and healthy food accessibility • Race/ethnicity/culture/language and socioeconomic status	Low-income neighborhoods had greater exposure to fast food outlets and convenience stores and fewer resources for reaching major food retailers. The Indigenous populations were 2.68 times more likely to have a proximate fast food outlet. Low income could be a significant predictor of exposure to unhealthy foods, which may contribute to the high prevalence of obesity among Indigenous populations.
Wenman (45)	• Indigenous (*n*=70) and non-Indigenous (*n*=1,905) pregnant women in a city in Alberta • The association between prenatal risk factors and birth outcomes • Place of residence, race/ethnicity/culture/language and gender	Indigenous participants were more likely to have babies with excessive birth weight. After considering that Indigenous women had higher prenatal risk factors (smoking: 41% vs. 13%; cervicovaginal infection: 33% vs. 13%; low income: 32% vs. 9%), the ethnic variable lost statistical significance. Behavioral factors, health status, and income level were associated with the negative birth outcomes among Indigenous women.
Employment		
Templeton (46)	• Low-income Indigenous families in Alberta (*n*=218) • Social determinants of health • Race/ethnicity/culture/language, gender and socioeconomic status	Approximately 75% of family caretakers were currently unemployed. The mean age of the youngest child of these unemployed caretakers was much younger compared to employed caretakers (3.7 years vs. 5.8 years). The parents might wait until the youngest child becomes of school-age before seeking employment; childcare may restrict employment, especially for single parent households or female caretakers. The caretakers commonly worked as cashier, food and beverage server, administrative assistant, house cleaner/housekeeping attendant, and child and youth care worker.
Alberta Human Services (47)	• Entire Indigenous Albertans (Census data from Statistics Canada) • Employment status • Race/ethnicity/culture/language	Compared to non-Indigenous populations, Indigenous Albertans had lower participation rate and employment rate (67.7% vs. 73.3%; 60.2% vs. 69.7%, respectively) and higher unemployment rate (11.1% vs. 5.5%). Métis peoples had the highest employment rates (62.6%) among Indigenous groups. Over half of participants worked in the following four industries: trade (17.4%); construction (12.2%); forestry, fishing, mining, oil, and gas (10.9%); and healthcare and social assistance (9.8%).
Lachance (4)	• First Nations communities in Alberta (*n*=45) • Participant in labor force, employment rate, and unemployment rate • Place of residence, race/ethnicity/culture/language and education	62% of First Nations in Alberta and 74.4% of non-First Nations in Alberta participated in labor force (a significant gap existed). A significant variation of labor force participation rate among First Nations communities could not be explained by geography. Unemployment rate was higher in First Nations populations than non-First Nations populations. 52.5% of First Nations in Alberta and 71.5% of non-First Nations in Alberta were working. The employment rate increased with higher education attainment. For First Nations, the employment rate was 36.1% if high school had not been completed, 65.1% if completed, and 78.1% if graduated from university.
Employment and education		
Miller (48)	• Homeless youths including Indigenous youth in two cities in Alberta (*n*=41) • Experiences of being homeless or at risk of being homeless • Place of residence, race/ethnicity/culture/language, socioeconomic status and Plus (age)	Most participants were not attending schools, and left school before high school graduation. The participants perceived that limited educational attainment was a major barrier to employment, which resulted in low income, homelessness, and poor quality of life in a cyclical manner. Higher education was associated with higher employment rates and income, and better housing opportunities.
Housing		
Lachance (4)	• First Nations communities in Alberta (*n*=45) • Proportions of households living in a crowded house and in a house requiring major repairs • Place of residence and race/ethnicity/culture/language	17.7% of First Nations in Alberta and a third of on-reserve First Nations in Alberta lived in a crowded house (<5% of the total provincial and national populations). 30.6% of First Nations in Alberta and 28.6% of First Nations in Canada lived in houses requiring major repairs (6% of non-First Nations in Alberta and 7% of non-First Nations in Canada).
Wearmouth (49)	• First Nations peoples with spinal cord injuries in Alberta (*n*=7) • Experiences of mobility and accessibility on reserves • Race/ethnicity/culture/language	The participants’ dwellings and community facilities were not properly equipped to accommodate the mobility needs of these populations.
Belanger (50)	• Indigenous peoples in a city in Alberta (*n*=1,068) • Housing needs • Place of residence and race/ethnicity/culture/language	Of all surveyed households, 15.7% needed major repairs, and 54.3% required minor repairs; 23.3% were living in crowded dwellings; 57.1% perceived that appropriate housing was less affordable due to low income; only 6.7% were eligible for a mortgage to buy a house with an average price of $270,000.
King Blood (51)	• First Nations peoples who live on-reserve (*n*=1,322) • Social determinants of health • Place of residence and race/ethnicity/culture/language	Dwelling conditions on reserves were generally poor. In 2002/03, the participants lived in houses without smoke detectors or fire extinguishers (50%) and in crowded dwellings (32%).
Education		
Alberta Education (52)	• Entire students including Indigenous students in Alberta • Overview of educational outcomes in Alberta • Race/ethnicity/culture/language	High school drop-out rates for Indigenous students declined from 11.8% in 2006 to 9.0% in 2010.
McKennitt (53)	• Indigenous children from two elementary schools in a mid-sized city in Alberta (*n*=18) • Effect of culturally sensitivity on smoking prevention program • Place of residence and race/ethnicity/culture/language	Culturally sensitive smoking prevention programs may effectively reduce future smoking intentions among Indigenous children.
Pigford (54)	• 4th- and 5th- grade First Nations students in Alberta (*n*=15) • Importance of including opinions of First Nations children for health promotion programs • Race/ethnicity/culture/language	A limited understanding about young learners’ educational preferences, needs, and aspirations, as well as cultural specificity in health promotion programs may result in less than ideal knowledge translation.
Kulig (55)	• Indigenous nursing students and educators of the University of Lethbridge (*n*=85) • Strategies for promoting educational attainment among Indigenous students within post-secondary educational settings • Race/ethnicity/culture/language	Culturally relevant and appropriate pedagogical approaches for Indigenous students (e.g. mentorship and transitioning programs) were suggested.
Lachance (4)	• First Nations communities in Alberta (*n*=45) • Educational attainment across First Nations communities • Place of residence and Race/ethnicity/culture/language	51.9% of First Nations in Alberta, and 64.1% of on-reserve First Nations have not completed high school (39.1% of First Nations in Manitoba, 48.4% of First Nations in Canada and 23.8% of Canadian population have not completed high school). By the age of 34, a third of First Nations in Alberta, and 57% of on-reserve First Nations have not completed high school. First Nations are three to five times more likely to have not completed high school compared to non-First Nations in Alberta. Educational attainment was significantly different across communities.
Wishart (56)	• An inner-city high school for Indigenous students in a city in Alberta • Strategies for promoting educational attainment among urban Indigenous early school leavers • Place of residence and race/ethnicity/culture/language	There was a gap between Indigenous and Western cultures, worldviews, and knowledge in curriculum, policies, and pedagogical practices. Involving Elders in Indigenous education was suggested.
Ralph-Campbell (57)	• Indigenous peoples with diabetes in Alberta (*n*=394) • The association between education and prevalence of diabetes • Race/ethnicity/culture/language and education	The lower level of education was associated with the high prevalence of diabetes among Indigenous participants.
Virani (58)	• First Nations peoples with diabetes from 44 First Nations communities in Alberta (*n*=1,151) • Rationale and implementation a chronic disease prevention program • Place of residence and race/ethnicity/culture/language	There were modest improvements in some program outcomes of a chronic disease prevention program. Community acceptance played a significant role in successful implementation of the program.
Toth (59)	• 44 First Nations communities in Alberta • Ways of addressing the diabetes epidemic in Indigenous communities • Place of residence and race/ethnicity/culture/language	Community-based and community-driven health promotion approach involving knowledge users, beneficiaries, and stakeholders of communities in developing and implementing a program was one of the key factors for successful intervention.

## Results

**Fig. 1:**
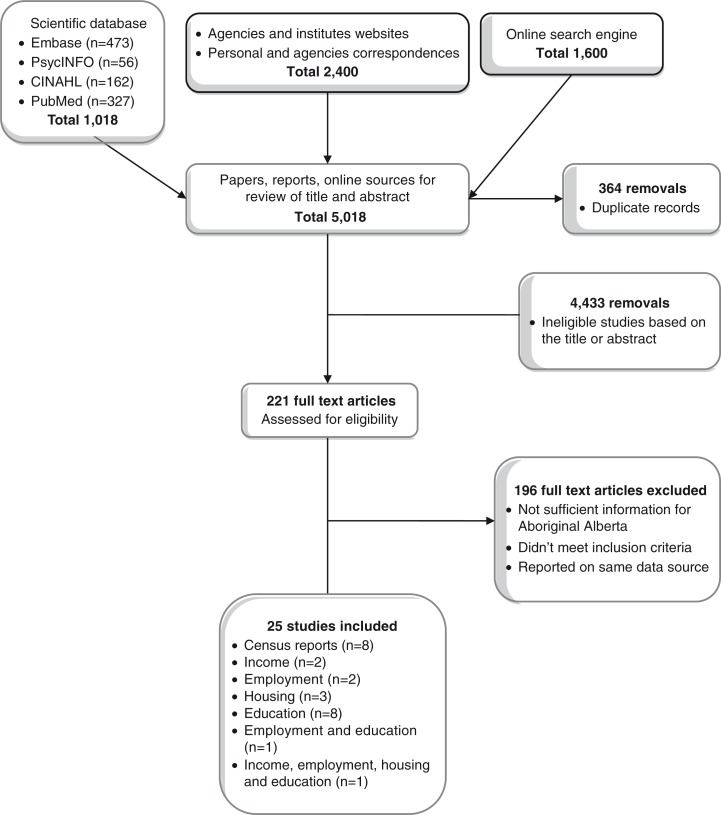
Search results and number of excluded and included studies in the systematic review on four major social determinants of health (income, education, employment, and housing) among Indigenous populations in Alberta.

A total of 5,018 records were identified by searching several scientific databases (*n*=1,018), examining agency and institute websites and correspondence (*n*=2,400), and consulting online search engines (*n*=1,600). After removing duplicates (*n*=364) and screening the remaining 4,654 titles and abstracts, 4,433 were excluded based on a lack of information on the four major SDH among the Indigenous populations in the title and/or abstract. The remaining studies (*n*=221) were reviewed in full-text to further determine their eligibility. After the full-text review, a total of 24 sources were included: eight census reports, two studies on *income*, two studies on *employment*, three on *housing*, eight studies on *education*, one on both *employment and education*, and one on all four SDH (Fig. 1).

### Income

On average, Indigenous peoples in Alberta had lower income levels, higher poverty rates, and a higher dependency on financial aids from governments compared to non-Indigenous populations in 2011 (Table 1). In 2006, the average income of Indigenous populations (i.e. total income of individuals aged 15 years and over) in Alberta was lower than that of non-Indigenous populations in the province ($29,466 vs. $42,998) (36–41). The median household income (after-tax) ranged between $17,056 and $50,944 for First Nations Treaty communities (non-First Nations community of southern and central Alberta was in the $50,000–$60,000 range; those of northern Alberta had a higher median, as high as $97,483); the source recognized that these disparities might have an association with geography, family status, educational attainment, and literacy level (4).

In 2011, this income trend continued. For Indigenous populations, average income ($35,437 vs. $50,956), average employment income ($55,668 vs. $69,438), the total income of individuals aged 15 years and over who worked full-time all year and average household income ($83,437 vs. $100,819) were lower than the income of non-Indigenous populations (42). The income level of Indigenous peoples in Alberta, however, was higher than the national level ($35,437 vs. $29,780) (42). The percentage of low-income families among Indigenous populations was almost double that of non-Indigenous populations (20.6% vs. 10.7%) (42). This provincial percentage had increased since the 2006 Census (18.7%), but was lower than the national average for Indigenous Canadians (25.3%) (36–42). Female Indigenous populations had lower incomes than male ($27,871 vs. $43,601) (42).

Rates of dependency on government transfers (e.g. child benefits and tax rebates) were higher among Indigenous peoples in Alberta (12.9%) than among non-Indigenous populations (7.3%) (42), but lower than the national average for Indigenous Canadians (18.5%). The highest dependency rates were found in communities within Treaty six territories, whereas more remote communities within Treaty eight lands have some of the lowest dependency rates among Indigenous communities (4).

We retrieved two studies regarding income and its impacts on the health and well-being of Indigenous Albertans (Table 2). A study of the accessibility of healthy foods within 215 residential neighborhoods in Edmonton showed that neighborhoods with low incomes had a greater exposure to fast food outlets and convenience stores and fewer resources for reaching supermarkets or major food retailers. In particular, the study indicated that the Indigenous populations were 2.7 times more likely to have a proximate fast food outlet. Authors suggested that income level could be a significant predictor of exposure to unhealthy foods, which might contribute to the higher prevalence of obesity (44).

A study of the association between prenatal risk factors and birth outcomes revealed that Indigenous participants were more likely to have babies with heavier birth weight. However, ethnicity was not solely associated with this possibly adverse neonatal outcome. After adjusting for higher prenatal risk factors for Indigenous women compared to non-Indigenous women—that is, smoking (41% vs. 13%), the presence of cervicovaginal infection (33% vs. 13%), and low income (32% v. 9%)—the ethnic variable lost statistical significance. Behavioral factors, health status, and income level were associated with the birth outcomes among Indigenous women (45).

### Employment

Indigenous peoples in Alberta had lower participation (i.e. the ratio of individuals who were either employed or were actively seeking employment) and employment rates, and had a higher unemployment rate compared to non-Indigenous peoples in 2011 (Table 1). The participation rate for Indigenous Albertans was 6% lower than that for non-Indigenous populations (42). Unemployment among Indigenous Albertans was two times higher than among non-Indigenous peoples (11.1% vs. 5.5%), and was higher among males compared to females (12.7% vs. 9.2%) (42). The employment rate among Indigenous Albertans was 60.2%, which was 9.5% less than among non-Indigenous Albertans, and was higher among males compared to females (42). Other statistics showed that Métis peoples had the highest employment rate at 62.6% among Indigenous groups (47), and that unemployment and employment rates varied significantly by First Nations communities (data not shown) (4). There was no significant difference in these employment measures between the years 2006 and 2011 (36–42).

In 2011, more than half of Indigenous Albertans worked in the following four industries: trade (17.4%); construction (12.2%); forestry, fishing, mining, oil, and gas (10.9%); and healthcare and social assistance (9.8%) (47). The most commonly reported job titles were cashier, food and beverage server, administrative assistant, house cleaner/housekeeping attendant, and child and youth care worker (46).

It appeared individuals’ circumstances (i.e. personal and social life contexts) and socioeconomic positions (e.g. gender and education) influenced the employment status among Indigenous Albertans. A study of SDH among 207 low-income families indicated that approximately three-quarters of caretakers within these families were not currently employed. The mean age of the youngest child of these unemployed caretakers was much younger (3.7 years), compared to those caretakers who were employed (5.8 years). The authors speculated that the parents may wait until the youngest child becomes of school-age before seeking employment, and that childcare might be a barrier to employment, especially for single parent households or female caretakers (46). Another study of homeless individuals, including Indigenous youth, reported that low educational attainment and income, in addition to a high rate of unemployment and homelessness, were cyclically related and negatively affected quality of life (48).

### Housing

Sources indicated that Indigenous peoples in Alberta were more likely to live in dwellings with inappropriate housing conditions and private spaces and to have a limited affordability for dwellings (Table 1). In 2011, the proportion of Indigenous Albertans who lived in dwellings requiring major repairs (i.e. repairs to plumbing, electrical wiring, and/or structural maintenance to walls, floors, or ceilings), was significantly higher than that of non-Indigenous Albertans (16.2% vs. 6.4%) (42). Over 30% of on-reserve First Nations peoples lived in dwellings requiring major repairs in 2006 (4), and approximately 50% of the populations lived in houses without smoke detectors or fire extinguishers (51). Among Métis Albertans, 14% lived in dwellings requiring major repair, and a greater proportion of rural houses were in poor condition than houses in urban areas (19% vs. 10%) (36–41). A case study of housing conditions in a city in Alberta showed that 15.7% of the houses needed major repairs, and 54.3% of houses required minor repairs (50). Another study of on-reserve First Nations peoples with physical disabilities reported that the participants’ dwellings and community facilities were not properly equipped to accommodate the mobility needs of these populations (49).

Crowded dwellings indicate limited private spaces within a household dwelling. They are characterized as houses with more than one individual per room (including kitchen, bedroom, and living room, but excluding hall, bathroom, laundry room, and attached shed) (4). Living in crowded housing was more common among Indigenous Albertans than among non-Indigenous Albertans (4.2% vs. 1.7%) (42). Likewise, the prevalence of crowded dwellings was high among First Nations peoples (17.7%), and almost one-third of the peoples who lived on-reserve were living in crowded houses (4). Another study showed that 32.1% of First Nations families with children lived in crowded dwellings (51). Among Métis Albertans, 5% were living in crowded dwelling conditions, and about 8% who lived in rural areas experienced the same challenges (36–41). Approximately one in four surveyed households in a city in Alberta were living in crowded dwellings (50).

In 2011, the proportion of Indigenous tenant-households (i.e. renters) who spent 30% or more of household total income on shelter costs (rental fee and utility payments) was 43.4% (42). Over half of the participants in a city in Alberta perceived that appropriate housing was difficult to afford and only 6.7% of the participants were eligible for a mortgage to buy a house with an average price of $270,000 (50).

### Education

There was a large gap in educational achievement between Indigenous and non-Indigenous populations aged 15 years and above who lived in Alberta in 2011 (Table 1). Approximately 40% of Indigenous Albertans did not complete a high school education (42). High school completion rates were also lower among Indigenous Albertans than among non-Indigenous Albertans (24.2% vs. 26.6%) (42). While 55.5% of non-Indigenous Albertans attained some form of post-secondary education, only 36.0% of Indigenous Albertans did so (42). In addition, Indigenous Albertans were 3.5 times less likely than non-Indigenous Albertans to complete a university degree (6.2% vs. 21.7%) (42).

The 2006 census revealed educational inequities among First Nations populations in Alberta. About 64% of off-reserve and 52% of on-reserve First Nations peoples had not completed high school (4, 36–41). The rates were significantly higher than those of non-Indigenous Albertans (22.3%), First Nations populations in Canada (48.4%), and First Nations populations in other provinces (e.g. Manitoba at 39.1%) (4).

Educational attainment for Indigenous populations has, however, improved over the years. Between 2006 and 2011, the proportion of peoples who did not complete high school decreased by 4.5%, and high school and post-secondary completion increased by 2.8 and 2.7%, respectively (36–42). High school drop-out rates for Indigenous students have also shown a steady decline from 11.8% in 2006 to 9.0% in 2010 (52). Indigenous students appeared to be remaining longer in school. The top five major fields of study were architecture, engineering, and related technologies (23.1%); business, management, and public administration (13.9%); health and related fields (10.6%); personal, protective, and transportation services (7.7%); and social and behavioral science and law (7.2%) in 2011 (42).

These trends were especially positive, given that higher education is associated with higher employment rates and income levels, and better housing opportunities (4, 48). Employment for Indigenous Albertans increased significantly with higher levels of education (4). In 2006, the employment rates for First Nations peoples who had not completed high school was 36.1%, while the rates for those who had completed high school and university degree programs were 65.1 and 78.1%, respectively (4). Education also significantly impacted the income level of Indigenous Albertans (4). The median income was 50% higher among those who had completed high school than those who had not (4). The difference in lifetime income between First Nations peoples without high school diplomas and those with university degrees could be over $750,000 (4). A qualitative study with homeless Indigenous youth reported that a small number of the participants were attending schools, but the majority had left school prior to high school graduation. The participants perceived that educational attainment was a major barrier to employment, which might have resulted in a low income level and homelessness (48).

A lower level of education was associated with the high prevalence of type 2 diabetes among Indigenous population in northern Alberta (57). Limited culturally-specific health educational opportunities are available for Indigenous Albertans. For example, a qualitative study on a First Nations community stated that there had been a lack of understanding about young learners’ educational preferences, needs, and aspirations (54). Accordingly, there had been a considerable gap between academic expertise and community knowledge within community-level health education among Indigenous Albertans (54). This study indicated that an insufficient cultural specificity had resulted in improper and disconnected knowledge translation (54).

Two studies revealed a gap between Indigenous and Western cultures, worldviews, and knowledge in provincial curriculum, policies, and pedagogical practices (55, 56). To promote educational attainment for urban Indigenous students, authors suggested 1) the involvement of Elders in Indigenous education and 2) culturally relevant and appropriate educational approaches such as mentorship and transitioning programs for Indigenous students (55, 56).

Community-based health education programs, such as smoking prevention for Indigenous students (53) and the SLICK project (Screening for Limb, I-Eye, Cardiovascular and Kidney Complications in Individuals with Type 2 Diabetes) (58, 59), also demonstrated the possibilities of successful interventions through community-based and community-driven health education programs that focus on the participation of knowledge users and beneficiaries, as well as stakeholders, in the development and implementation process (53, 58, 59).

## Discussion

Our review emphasized that there are disparities in SDH and current health and well-being among Indigenous peoples in Alberta. Accordingly, there is a need to identify practical strategies for 1) promoting resiliency against residual effects of trauma and 2) incorporating Indigenous culture and contextual factors affecting Indigenous health to ensure current policies are culturally appropriate and promote positive public perceptions. Understanding population-specific circumstances is crucial in developing and implementing future health-related policies and actions. As such, researchers and policy-makers should carefully examine population-specific determinants of health and their implications while engaging in and developing scholarly and professional Indigenous initiatives.

### Income disparities and health

In general, individuals with limited income experience higher prevalence of acute and chronic diseases (60–63); and also tend to participate less in screening services for chronic diseases, and experience subsequent burdens and disadvantages from having a greater risk of chronic diseases, a higher incidence of chronic diseases, and a higher mortality rate (64–69), such as shorter life expectancy and lower survival rates with age (70). The results of our review regarding income and its impacts on the health and well-being of Indigenous Albertans highlighted the associations between income and material determinants of health (i.e. the accessibility of healthy foods), and biological determinants of health (i.e. birth outcomes). An important aspect in income and economy of Indigenous populations is that traditional economic activities or sources of income, such as trapping, hunting, and crafting are often not counted as income or employment in census data (71, 72) and are difficult to capture.

There is limited information on income status among different age groups, especially youths and seniors; Indigenous ethnicities (e.g. First Nations, Métis, and Inuit); and dwelling areas (e.g. urban, rural, and on-reserve). Accordingly, further investigation is needed to understand age, ethnicity, and place-related income status and its implications for health and wellness among Indigenous peoples. Furthermore, associations amongst income, personal and social circumstances (e.g. family composition, remoteness of healthcare facilities, and income support dependency), and health outcomes should be examined thoroughly to develop and implement future income-related policies and actions. Finally, qualitative studies are required for an in-depth exploration of the barriers to, and opportunities for, the elimination of income inequity amongst Indigenous populations.

### Employment disparities and health

Studies have indicated that unemployment is closely linked to health inequity (4, 24, 73–75). The adverse impacts of unemployment on health outcomes include greater risks of chronic diseases, cardiovascular mortalities, and mental health challenges (76–79). Unemployment has also been associated with unhealthy and high-risk lifestyles such as smoking, alcohol and substance abuse, unhealthy diets, physical inactivity, and domestic violence (80–83); a relatively high unemployment rate in Indigenous populations in Alberta may pose these health risks. A study included in this review suggested childcare as a barrier to employment, particularly for single parents or female caretakers (46). Overall, women are more likely than men to give up employment to take care of family members (84). Employment status and socioeconomic status may be challenging to the mental health of single mothers (85). Given that Indigenous women tend to have more children, be single parents, and have more family members compared to non-Indigenous women (86). In addition to the Indigenous-specific SDH described in this review, particular attention is needed when considering policy development. Unfortunately, there is limited information about the role of gender in family care, employment, and related health among Indigenous populations in Canada.

Our review also identified a knowledge gap regarding the ways in which social capital and cohesion (e.g. social relationships, communication, respect, and trust) influence employment among Indigenous populations. A national survey indicated that incidence rates of workplace racism and discrimination were higher among Indigenous employees (42.3%) than non-Indigenous employees (33.5%) (87). As such, further investigation is required for an in-depth understanding of the role of social capital and cohesion in employment amongst Indigenous populations, and to identify strategies for improving these factors as a way of reducing unemployment rates. Moreover, as mentioned in our review, a substantial proportion of the Aboriginal populations in Alberta is involved in trade, construction, forestry, fishing, mining, oil, and gas industries (47), which may expose employees at a risk of workplace injury. A study conducted in the province of British Columbia, Canada, concluded that Aboriginal identity predicted workplace injury risk and that culturally appropriate programs to address the issue were needed (88).

To inform future unemployment-reduction planning and decision-making among Indigenous peoples, expanded studies should be undertaken in various related areas, including 1) the root causes of employment disparities among diverse Indigenous population groups (e.g. females, low-income families, and families with young children); 2) the implications of public policies and social supports, behavioral factors (e.g. high-risk behaviors such as smoking), and psycho-social factors (e.g. interpersonal skills and resiliency) for employment; and 3) employment status and its impacts on the health and well-being of the populations (e.g. the mental health consequences of unemployment or exposure to workplace injury).

### Housing disparities and health

Appropriate housing can be identified on the grounds of adequate daily living facilities and equipment, sufficient numbers of private living spaces, and affordability (4). Several studies have revealed that inappropriate housing and homelessness are associated with high-risk behaviors (89–92) and increased mortality rates (93). According to a longitudinal study, the life expectancy of homeless males at age 25 years is 10 years lower, and survival rates between ages 25 years and 75 years were 31.9% lower compared to the general Canadian population (93). Our review revealed that a higher proportion of Indigenous peoples in Alberta live in inappropriate housing conditions than non-Indigenous peoples. Also, the proportion and severity of poor housing condition was greater in on-reserve communities. Inappropriate dwelling conditions and quality are associated with a number of health issues such as unintentional injuries, respiratory and infectious diseases, mental and psychological challenges, and domestic violence (4). However, we drew attention to the limited knowledge regarding 1) factors contributing to current housing status among Indigenous populations and 2) housing and its impacts on health outcomes. A limitation of our review was the shortage of peer reviewed studies on housing-related health challenges. In addition, there is insufficient information on housing status among different Indigenous population groups. Further investigation would be beneficial to understand fully the challenges related to housing inequity and the resulting health implications among Indigenous populations, as well as to inform future programs and policies in this area.

### Education disparities and health

Education can improve individuals’ skills and knowledge of ways to maintain, promote, and restore health and wellness, while lower level educational attainment is associated with health disparities and high mortality rates (63, 94–97). Tjepkema et al. reported that age-standardized mortality rates were highest among people who achieved less than a secondary level education and were lowest among post-secondary degree-holders (95). However, among 80% of Indigenous Albertans who previously experienced racial discrimination, about 66% had experienced high levels of racism while they were in schools (98). These students were two to three times more likely to be a victim of racial discrimination than a reference sample of other ethnic minority groups in the United States (98). Furthermore, students who practiced Indigenous traditions and cultures were more likely to experience racism (98). The participants reported that racial discrimination within school settings caused feelings of helplessness and hopelessness and experiences of shock and/or frustration (98).

To promote educational attainment among Indigenous peoples, governmental agencies and stakeholders have developed and implemented various educational policies and actions. For example, the Memorandum of Understanding for First Nations Education between Treaty Chiefs in Alberta and federal and provincial governments was established to promote educational outcomes among First Nations students (99). Furthermore, the First Nations, Métis and Inuit Education Policy Framework (100) and resources for classroom teachers and staff (101) have been utilized. Nationally, educational models and frameworks have also been suggested, including the Holistic Lifelong Learning Model for each First Nations, Métis, and Inuit population groups (102) and the Framework for Indigenous School Health (103). A small number of studies included in our review examined the effectiveness of a culturally appropriate approach to education of Indigenous students. Future studies should examine how these population-specific efforts to promote educational attainment impact the holistic view on health and health status.

There is limited information on educational status among genders, Indigenous ethnicities (First Nations, Metis, and Inuit), age groups (e.g. early learners such as toddlers, and elementary and junior high school students), and dwelling areas (e.g. urban and rural). Furthermore, there is a scarcity of scientific studies regarding the barriers to, and opportunities for, education among Indigenous populations (e.g. reasons for school drop-out, obstacles to participating in post-secondary education programs, and strategies for overcoming racial discrimination in educational settings). Finally, there is a shortage of studies on practical strategies for developing and implementing culturally relevant and appropriate health educational programs. Further investigation is needed in the following areas: 1) the identification of educational disparities for specific Indigenous populations, 2) strategies for overcoming educational barriers in diverse educational settings, 3) approaches to improve health literacy, the ability to access and effectively apply information to promote and maintain health among Indigenous peoples, and 4) maintaining the long-term effects of health education through culturally-specific approaches.

The four SDH in this paper are described specifically for Indigenous peoples in Alberta; however, some of the general Canadian population also experience these SDH (64, 104–106). What is unique for the former population is that many health-related issues appear to be largely attributable ‘collective’, ‘normalized’, and ‘structural or implicit (15)’ contextual factors. Veenstra discussed that when explaining health disparities among various ethnic groups not only should race or genetic predisposition be considered, but also experiences of racial discrimination (107). Similarly, Daoud et al. concluded that socioeconomic status alone did not fully explain a health behavior among Aboriginal women, and those contextual factors and related SDH factors should be taken into account (108). Since mid-1980, the Canadian government started recognizing the injustices experienced by Indigenous peoples. The subsequent policy of handing out financial compensation to the survivors of colonialism and residential schools for the traumatic experiences they faced, has proven insufficient. Many recipients felt the value did not compensate them adequately to overcome the sense of injustice of the negative health outcomes (9).

Traditionally, many researchers and practitioners have focused on identifying and addressing an individual's responses to traumatic events as major determinants of health for Indigenous populations (109), without giving careful consideration to the root causes associated with these individual responses (23, 109). Efforts to overcome the ongoing health inequities amongst Indigenous peoples should involve understanding the contextual determinants of health and the interactions between these broader systemic environments and individuals’ responses to these circumstances (15, 23, 109). Studies also recommend investigating ways of addressing unresolved past trauma or transgenerational trauma as a means of promoting health and well-being among survivors and descendants. This may include understanding the ways in which 1) Indigenous Canadians restore the lost sense of Indigenous identity (including languages, cultural traditions, and community practices), as well as recover and develop hope, resiliency, secure social connections, and interpersonal efficacy within Westernized and urbanized life contexts (7, 15, 16, 23, 110, 111); 2) professional practitioners and service providers who work with Indigenous populations establish a space that encourages and supports open communication, to gain insights from the perspectives of Indigenous Canadians in developing and implementing strategies for holistic, inclusive, and culturally appropriate health promotion (7, 23, 112); and 3) these professionals and stakeholders establish long-standing commitments to building trust and sustaining relationships (13, 23, 110).

Many Indigenous Canadians experience some common SDH, including the negative effects of colonization, and the loss of land and cultural resources. Racial discrimination and social exclusion are also often considered common experiences in Indigenous communities. However, contexts and circumstances are different among different Indigenous groups in various geographical locations, and the impacts of these varying factors have not been evenly experienced among Indigenous peoples (24, 113). Accordingly, particular considerations in understanding SDH for and within each Indigenous group, namely, First Nations, Métis, and Inuit, within a specific geographic fragment (e.g. provincial/territorial, urban, rural, on-reserve, off-reserve, etc.) and collaboration between various sectors to address complexity of SDH in Indigenous populations are suggested.

This systematic review contains some limitations. First, only studies and reports published in English were included in this review. However, a secondary search for papers published in French yielded nine scientific publications, the full-text of which was also available in English. Therefore, these nine publications had already been included in our original literature search. Second, the authors focused only on the four major SDH recognized by the Community Well-Being Index of Canada. Other SDH, such as gender and social capital, may also significantly impact the health of Indigenous populations in Alberta. Third, many studies included in this review focused on the current status of SDH among Indigenous Albertans, with less focus on the direct impact of the SDH on Indigenous health or possible strategies to lessen this impact. Lastly, there was limited information about SDH among subgroups within Indigenous populations (e.g. ethnicity – First Nations, Métis, and Inuit, age, gender, residential location, etc.). Overall, however, a robust search of available information, both scientific and grey literature, was conducted. The authors also identified current knowledge gaps specific to the province of Alberta and provided suggestions for decision makers. Furthermore, our review clearly reveals a need for more in-depth studies of Indigenous populations in Alberta.

## Conclusions

Our review highlighted some of the root causes of health disparities among Indigenous peoples, population-specific SDH frameworks and considerations in understanding the determinants of health among Indigenous populations, and knowledge gaps and areas requiring further investigation into the four major SDH for Indigenous populations. Future studies should seek to identify 1) the status of health and well-being among diverse Indigenous populations; 2) the root causes of health disparities and inequities for each group; and 3) other structural and implicit, as well as personal and subjective, factors affecting the health and well-being of Indigenous populations. Furthermore, based on current knowledge about health disparities among Indigenous populations, community-based intervention programs and actions should follow urgently to address these issues.
